# Factors influencing nurse fatigue during COVID-19: regression vs. fuzzy-set qualitative comparative analysis

**DOI:** 10.3389/fpubh.2023.1184702

**Published:** 2023-08-17

**Authors:** Huanyu Zhang, Zhixin Liu, Junping Liu, Yajie Feng, Dandan Zou, Juan Zhao, Chen Wang, Nan Wang, Xinru Liu, Lin Wu, Zhaoyue Liu, Libo Liang, Jie Liu

**Affiliations:** ^1^Department of Social Medicine, School of Public Health, Health Management College, Harbin Medical University, Harbin, China; ^2^Department of Health Policy and Management, School of Public Health, Peking University, Beijing, China; ^3^Jinshan Hospital of Fudan University, Shanghai, China; ^4^Southwest Hospital, Third Military Medical University (Army Medical University), Chongqing, China; ^5^Xinqiao Hospital, Third Military Medical University (Army Medical University), Chongqing, China; ^6^Second Affiliated Hospital of Harbin Medical University, Harbin, China

**Keywords:** fatigue, nurses, COVID-19, fsQCA, anxiety

## Abstract

**Background:**

Nurses during COVID-19 who face significant stress and high infection risk are prone to fatigue, affecting their health and quality of patient care. A cross- sectional study of 270 nurses who went to epidemic area to support anti-epidemic was carried out *via* online survey during the COVID-19 pandemic on November 2021.

**Methods:**

A web-based cross-sectional survey of 270 nurses in China who traveled to Heihe City in Heilongjiang Province to combat the novel coronavirus epidemic. The researchers collected information on sociodemographic variables, anxiety, transition shock, professionalism, collaboration, hours of work per day, and fatigue. Regression and fuzzy-set Quality Comparative Analysis (*fsQCA*) evaluated the factors’ impact on the nurses’ fatigue.

**Results:**

Regression analysis showed that the psychological variables significant for fatigue, transition shock (*β* = 0.687, *p* < 0.001) and anxiety (*β* = 0.757, *p* < 0.001) were positively associated with fatigue, professionalism (*β* = −0.216, *p* < 0.001) was negatively associated with fatigue, and among the work-related variables, cooperation (*β* = −0.262, *p* < 0.001) was negatively related to fatigue. FsQCA analysis showed that combined effects of work hours, anxiety, and nurses’ educational status caused most of the fatigue (raw coverage = 0.482, consistency = 0.896).

**Conclusion:**

This study provides two main findings, the one is the greater transition shock experienced during COVID-19 in a new environment, low levels of professionalism, anxiety, and poor nursing teamwork situations lead anti-epidemic nurses to increased fatigue. Second, the fsQCA results showed that anxiety is sufficient for fatigue and that nurses’ educational status, daily working hours, and anxiety are the most effective combination of factors.

## 1. Introduction

The novel coronavirus (COVID-19) is a highly transmissible and rapidly mutating virus that has spread worldwide since late 2019. Some people with the disease present with influenza-like symptoms, whereas others develop severe complications such as pneumonia, respiratory disorders, and even death (1). COVID-19 disrupts lives, impairs health, and creates stress, especially for healthcare workers, who face infection risk and the enormous workload of caring for patients. Among healthcare workers, nurses experience the most stress (2), including high workload, direct contact with infectious patients, uncertainty about treatment options and outcomes, dealing with patients’ families, and caring for critically ill and dying patients (3). In addition, high stress levels lead to many chronic physical illnesses and mental disorders, including severe fatigue.

*Fatigue* is a subjective discomfort that is not only a response to normal physiological conditions but also a clinical manifestation of certain diseases and is the primary cause of subfertility (4). Fatigue in nurses is a complex response to personal, unit, and health system demands (5). Although experiencing some fatigue while working is normal, excessive fatigue produces an overwhelming sense of exhaustion and decreased energy, which impairs physical and cognitive functioning (6), affecting the quality of life and well-being and leading to burnout and illness (7). In addition to adverse effects on the individual, fatigue affects work performance (6, 8), reduces productivity (9) and readiness to perform assigned duties (10), and increases medical errors (11). Nurses have a considerable impact on defusing a pandemic crisis. Their health status affects the provision of continuous and comprehensive care to patients and significantly affects how they respond to public health crises.

Studies show that many factors affect nurses’ fatigue. During the non-epidemic period, the effects of nurses’ sociodemographic characteristics were confirmed. Specifically, age and education affect nurses’ fatigue, with older workers likely to experience more severe fatigue processes because physical strength is limited by age. Furthermore, the higher their education level, the less frequently nurse fatigue occurs (6). In addition, certain psychological factors in nurses are conducive to fatigue. Nurses in a new and unfamiliar work environment can experience a lack of control over their work and feelings of insecurity (12). In particular, nurses engage in fast-paced, highly technically demanding work, long working hours, and many night shifts. These work conditions often entail life-threatening health risks and can produce and exacerbate fatigue (13). Some studies show that nurses given insufficient time to rest and recover, confronting longer working hours and irregular shift rules (6), have a higher risk of fatigue (14). They exhibit significantly increased error rates and injuries when their shifts exceed 12 consecutive hours (15). In particular, during the pandemic, nurses face elevated infection risk. In addition, the COVID-19 patient care workload is substantial. Nurses lack sufficient time to rest. The pressure they bear is likely to lead to fatigue (16). In contrast, professional collaboration not only improves nursing competence and maintains an environmental work atmosphere but also allows nurses to feel psychologically safe, receive higher levels of collegial support, and avoid excessive emotionality. Collaboration can help them to manage problems that arise in clinical work more effectively through collaboration and alleviate fatigue (17).

Many studies have been conducted on nurses’ fatigue in daily life (18, 19), but studies analyzing the combined effects of factors influencing nurses’ fatigue during COVID-19 are lacking. In addition, few studies have been conducted to determine which combined factors could contribute to fatigue. Studying the combined effects of different influencing factors can provide a new and integrated perspective on nursing practice. Therefore, it is essential to assess the combined effect of factors influencing fatigue in nurses during COVID-19. This study focused on assessing how various factors (socio-demographic variables, anxiety, transition shock, professionalism, teamwork situation, and daily work hours) influenced nurses’ fatigue using two different methods: regression modeling and fuzzy set qualitative comparative analysis (fsQCA). The results of this study may provide evidence and ideas to help nurses better deal with fatigue by identifying single and combined effects, with implications for policymakers, managers, medical staff, and researchers to develop and implement strategies.

## 2. Methods

### 2.1. Study design, setting, and sample

The study design was quantitative and cross-sectional, using an online survey. The study administered a questionnaire in November 2021 to nurses who supported anti-epidemic work in Heihe City, Heilongjiang Province, China, which included basic information and eight sections, including teamwork, fatigue, anxiety, and professionalism. The exclusion criteria were nurses working in different hospital departments where COVID-19 patients were admitted. Among these were those working directly with COVID-19 patients and nurses dealing with administrative matters.

### 2.2. Data collection

With consent, the researcher commissioned a nurse to release the questionnaire content of this study to the we-chat groups of approximately 500 nurses. All the we-chat groups were working groups of anti-epidemic nurses. Nurses from multiple hospitals and departments answered the questionnaire through an online questionnaire webpage. Data were collected through an online survey conducted in November 2021. During the survey, 270 responses were received and analyzed.

### 2.3. Measures

#### 2.3.1. Dependent variable

##### 2.3.1.1. Fatigue

Fatigue was assessed using the Chinese version of the Multidimensional Fatigue Inventory-20 (MFI-20) (20). The MFI-20 is a 20-item scale with five dimensions, namely, *General Fatigue (GF), Physical Fatigue (PF), Mental Fatigue (MF), Reduced Motivation (RM)*, and *Reduced Activity (RA)*. Each dimension contains two positive (e.g., “I feel very active”) and two negative items (e.g., “I tire easily”), scored using a 5-point Likert scale with a total score of 20–100, with higher scores representing higher levels of fatigue. The scale contains four common factors: physical fatigue, mental fatigue, decreased motivation, and decreased activity. The Chinese version of the MFI-20 scale has good validity and reliability (21). The four factors explained 56.852% of the variance cumulatively, and the 20-item discrimination index ranged from 0.262 to 0.750. The internal consistency of the overall scale was 0.882, and the internal consistency coefficients of the four factors (*physical fatigue, mental fatigue, decreased motivation*, and *decreased activity*) were 0.867, 0.776, 0.476, and 0.687, respectively.

#### 2.3.2. Independent variable

##### 2.3.2.1. Professionalism

Professionalism was assessed using the Chinese version of the Professionalism Attitude Inventory (22, 23). The original scale was developed by Professor Hall and revised by Snizek. The scale asks respondents about their perceptions of their current occupations, such as “I like my current job more than other things.” The scale includes 12 items. The same 5-point Likert scale is used as the original scale, with “1” representing *strongly disagree* and “5” representing *strongly agree*. The scale’s internal consistency was good (Cronbach’s *α* = 0.946), and a common factor was extracted using principal component analysis. In addition, the factor loadings of each item on the corresponding factor were greater than 0.4, and the cumulative contribution rate was 64.9%, indicating that the validity of the questionnaire was good.

##### 2.3.2.2. Transition shock

Transition shock was assessed using an adaptation of the Transition Shock Scale (24). You-ru Xue developed the scale. In this study, some irrelevant questions were removed to leave five questions, such as “I am too tired to do anything after work” and “It is difficult to predict what will happen at work.” The same 5-point Likert scale as the original scale was used, 1–5 representing *strongly disagree* to *strongly agree*. The total score ranged from 5 to 25, with higher scores indicating greater transition shock. The questionnaire’s reliability was good (Cronbach’s *α* = 0.811) with a Kaiser-Meyer-Olkin value of 0.835. A common factor was extracted using principal component analysis. The loadings for each question on the corresponding factor were greater than 0.4, with a cumulative contribution of 60.3%, indicating good validity of the questionnaire.

##### 2.3.2.3. Anxiety

The Generalized Anxiety Disorder Scale (GAD-7) was used to assess anxiety. Seven items on a scale of 0 to 3 are used to assess symptoms in the last 2 weeks, corresponding to not at all, a few days (*mild symptoms*), more than half of the time (*moderate symptoms*), and almost every day (*severe symptoms*). The overall score ranges from 7 to 28; higher scores indicate increased anxiety (25). The scale has good reliability and validity. Cronbach’s alpha was 0.898, indicating good internal consistency. The sensitivity, specificity, and kappa values of the GAD-7 were 86.2, 95.5%, and 0.825, respectively, suggesting that the GAD-7 has good validity.

##### 2.3.2.4. Cooperation

Cooperation was assessed with the Chinese version of the Assessment of Interprofessional Team Collaboration Scale (26, 27), which has three dimensions: partnership (8 items), teamwork (8 items), and team coordination (7 items), with a total of 23 items. Items are scored on a 5-point Likert scale, with 1 = never, 2 = rarely, 3 = sometimes, 4 = often, and 5 = always, with a total score ranging from 23 to 115. The internal consistency of the total scale was good (Cronbach’s *α* = 0.88). Cronbach’s alpha coefficients for each dimension ranged from 0.88 to 0.90, indicating good reliability.

##### 2.3.2.5. Sociodemographic variables

This study measured the person-specific factors of age and education level. Age was coded as a continuous variable. Education level was assessed as follows: “Associate’s degree and below,” and “Bachelor degree or above.”

### 2.4. Statistical analysis

Two methodologies were applied to analyze the influence of different variables on nurses’ fatigue: regression models and fsQCA. Regression models are suitable for analyzing ordered continuous variables. They can predict the relationships between independent and dependent variables. In contrast, fsQCA considers more causal conditions and combinations of paths related to the outcome variable and does not focus on the individual effects of each condition (28).

Regression models were used in the first step to identify influential variables from personal factors (age and education), psychological factors (anxiety, transition shock, and professionalism), and work-related factors (teamwork and work hours). Personal factors were entered in step 1, followed by psychological factors in step 2, and work-related variables in step 3. The regression analysis phase identified the independent variables significantly associated with fatigue. All variables were also analyzed for combined effects in the fsQCA stage to observe the combined utility of different factors on fatigue and to compare the differences between the two methods and factors influencing nurses’ fatigue.

Regression concerns correlations between variables, whereas qualitative comparative analysis (QCA) concerns explaining the overall relationship between sets. Therefore, QCA is suitable for small sample populations, and fsQCA is a type of QCA that explores combinations of influencing factors rather than individual ones (29). The operation of fsQCA is to first select the calibration conditions of variables according to a specified theory or experience for calibration to make each variable in the range of 0–1 so that the original measurement has an interpretable collective meaning. An analysis of necessary and sufficient conditions to produce the outcome is performed. Necessary conditions are those that must be present for an outcome to occur, while sufficient conditions refer to those conditions that might lead to an outcome and that may not be present. Results are then simplified by constructing truth tables to produce intermediate (only including logical remainders backed by theoretical or practical knowledge), parsimonious (using both configurations with actual observed cases and incorporating all “easy” and “hard” “logical remainders”), and complex solutions (complex solutions only analyze configurations with actual observed cases), to reveal the variables complex causal relationships to the quantity (30). In the analysis result, the symbol “*” means “and” and “~” means “not.” Raw coverage indicates the proportion of cases that can be explained by the combination of conditions. It is generally used to examine the strength of the explanatory power of the combination of conditions. Unique coverage indicates how many cases can be explained by the combination path only, which can also be described as the net explanatory power. In summary, the properties of fsQCA of the analysis combined effects apply to this study. Therefore this study used fsQCA to analyze the factors influencing nurses’ fatigue.

Specifically, in this study, age, education, anxiety, cooperation, professionalism, daily working hours, and the dependent variable (fatigue) were selected for fsQCA. QCA only works for data ranging from 0 to 1. Hence, it is necessary to calibrate the data collected from the field survey before conducting fsQCA. Based on suggestions proposed in previous research (31), this study calibrated the raw data of the selected variables. The calibration process is as follows: Three qualitative breakpoints (0.95, 0.5, and 0.05) were designated to calibrate the continuous variables (fatigue, cooperation, anxiety, transition shock, professionalism, and daily working hours). The higher it ranked, the closer to 1 in the calibration. Education was set to 0 or 1: “associate’s degree and below” was calibrated as “0.” “Bachelor’s degree or above” was calibrated as “1.” Considering the sample size of 270 in this study, the minimum number of cases required to extract a solution should be greater than one. Therefore, two was chosen as the minimum number of cases. In addition, the minimum consistency level threshold required for a particular solution to be meaningful was set at 0.75. Later, the relationship between these seven factors and fatigue was determined by necessity and sufficiency analysis and coverage measures (32, 33).

Means, frequencies, and standard deviations were used to present the descriptive data. Then, correlations between key variables were analyzed using Stata 17.0. Next, the effects of various variables on fatigue were examined using regression analysis. Finally, the level of fatigue significance was set at *p* < 0.05. The fsQCA was conducted using FSQCA 2.5.

### 2.5. Ethical considerations

The beginning of the questionnaire stated that the survey was anonymous and voluntary and that nurses could choose whether to participate. Relevant ethical approval for the original systematic review was obtained from the authors’ institution.

## 3. Results

### 3.1. Sample characteristics

Table 1 presents the demographic characteristics of the sample. The sample comprised 270 nurses aged 22–53 from the Heilongjiang province in China. Roughly 91 % (91.5%) of the respondents were women, and 8.5% were men. Most respondents had a bachelor’s degree or higher (89.3%). Finally, 235 (87.0%) respondents had an intermediate title. See Table 1 for detailed information.

**Table 1:** Descriptive statistics of participants’ characteristics (*N* = 270).

Characteristic	Category	*N* (%)
Age	≤30	99 (36.7)
	31–40	145 (53.7)
	>40	26 (9.6)
Gender	Male	23 (8.5)
	Female	247 (91.5)
Education level	Associate’s degree and below	29 (10.7)
	Bachelor degree or above	241 (89.3)
Marital status	Unmarried	65 (24.1)
	Married	191 (70.7)
	Divorced	14 (5.2)
Title	Without titles or primary title	14 (5.2)
	Intermediate title	235 (87.0)
	Deputy high title or above	21 (7.8)

Table 2 shows the primary variable descriptors and calibration values. The results showed that nurses had a more severe level of fatigue (mean value: 45.937). In terms of psychological factors, the level of professionalism was higher (mean value: 47.4481), anxiety was lower (mean value: 11.870), and nurses experienced a greater degree of transition shock (mean value:14.562). Regarding work factors, the level of teamwork was good (mean value: 92.088). However, large differences in teamwork scores between nurses (standard error:16.958) were found. Most nurses had to work about 5 h a day (mean value: 4.977). Table 2 shows additional details.

**Table 2:** Main descriptions and calibration values (*n* = 270).

	Age	Education	Daily working hours	Anxiety	Transformation shock	Cooperation	Professionalism	Fatigue
M	32.596	1.892	4.977	11.870	14.562	92.088	47.481	45.937
SD	5.858	0.310	2.465	5.193	4.304	16.958	8.941	12.924
MIN	22	1	4	7	5	23	12	27
MAX	53	2	20	28	25	115	60	100
Calibration values								
P5	44.2	2	4	7	7	63	32	26
P50	32	3	4	10	15	92	47	46
P95	24.8	3	8	21	21	115	60	65

M, mean; DT, standard deviation; Min, minimum; Max, maximum; P10, 10th percentile; P50, 50th percentile; P90, 90th percentile.

### 3.2. Results of regression analysis

The predictive influences of sociodemographic variables (age and education), psychological variables (anxiety, transition shock, and professionalism), and work-related variables (cooperation and hours worked per day) on fatigue were analyzed using regression. Three different steps were established: the first included sociodemographic variables (education and age), the second included psychological variables (anxiety, transition shock, and professionalism), and the last step included work-related variables (cooperation and hours worked per day).

As shown in Table 3, sociodemographic variables, namely, age and education, had a non-significant relationship with fatigue (*p* > 0.05). Of the psychological variables significant for fatigue, transition shock (*β* = 0.687, *p* < 0.001) and anxiety (*β* = 0.757, *p* < 0.001) were positively associated with fatigue, implying that the more severe the nurses’ shock and anxiety about the transition to the work environment, the more severe the fatigue. Professionalism (*β* = −0.216, *p* < 0.001) was negatively associated with fatigue, implying that nurses who have a strong sense of professional mission and believe that what they are doing is meaningful experience less fatigue to some extent. Among the work-related variables, cooperation (*β* = −0.262, *p* < 0.001) was negatively related to fatigue, implying that a good atmosphere of cooperation among nurses can reduce fatigue, whereas daily working hours (*β* = −0.007, *p* > 0.05) were not significantly related to fatigue.

**Table 3:** Regressions for the dimensions of sociodemographic, psychological, and work-related variables (*n* = 270).

Predictor variables	Dependent variable: fatigue					
	Step 1		Step 2		Step 3	
	*β*	*t*	*β*	*t*	*β*	*t*
Age	0.153	1.09	0.009	0.10	0.001	0.01
Education	−1.632	−0.62	−0.864	−0.46	0.105	0.01
Professionalism			−0.441^***^	−6.76	−0.216^***^	−3.16
Anxiety			0.813^***^	5.79	0.757^***^	5.46
Transition shock			0.895^***^	5.23	0.687^***^	4.25
Cooperation					−0.262^***^	−7.03
Daily working hours					−0.007	−0.03
Constant	42.379^***^	11.41	44.656^***^	8.26	61.325^***^	10.98
F test	0.523		52.96		51.70	
R-square	0.004		0.500		0.580	
△R^2^	0.004		0.496		0.080	

*means *p* <0.05, **means *p* < 0.01, ***means *p* < 0.001; n.s. represents non-significant.

### 3.3. Results of fsQCA

The results (Table 4) showed no necessary conditions for fatigue because the consistency is under 0.90 in all cases. Therefore, this study looked for potential configurations of these causal conditions that lead to fatigue (34). Table 4 presents the fsQCA results for configurations that produced sufficient conditions for fatigue, consistency, and coverage (including raw, unique, and solution coverage).

**Table 4:** Necessity analysis for Multidimensional fatigue.

	Fatigue		~Fatigue	
	Cons^*^	Cov^**^	Cons^*^	Cov^**^
Education	0.886	0.497	0.101	0.469
~Education	0.113	0.530	0.898	0.502
Age	0.624	0.656	0.612	0.640
~Age	0.657	0.630	0.670	0.640
Anxiety	0.935	0.639	0.746	0.507
~Anxiety	0.279	0.525	0.469	0.878
Transformation shock	0.677	0.707	0.594	0.618
~Transformation shock	0.634	0.611	0.71	0.689
Professionalism	0.570	0.539	0.511	0.542
~Professionalism	0.722	0.770	0.783	0.737
Cooperation	0.488	0.475	0.793	0.768
~Cooperation	0.762	0.787	0.458	0.471
Daily working hours	0.798	0.660	0.753	0.619
~Daily working hours	0.540	0.687	0.587	0.743

* consistency; ** coverage; condition required: consistency ≥ 0.90.

Table 4 shows that anxiety is necessary for fatigue as concordance exceeds 0.9 (33). Education, cooperation, professionalism, and daily working hours are sufficient conditions for fatigue, as the concordance is approximately 0.80; however, the explanatory power is weak, and the factors that influence fatigue in nurses should be analyzed in terms of a combination of variables.

Table 5 summarizes the three paths for fatigue according to the format outlined by Peer (30). In predicting fatigue, three paths were observed that explained 58.0% of the cases (Overall Consistency = 0.881; Overall Coverage = 0.580). This study makes the following key observations.

**Table 5:** Solution of case.

Frequency cutoff: 2	Fatigue		
	Consistency cut-off: 0.886		
	1	2	3
Age		⊗	⊗
Education	•		•
Professionalism	⊗	⊗	
Transition shock			●
Anxiety	●	●	●
Cooperation	⊗	⊗	⊗
Daily working hours	•	•	•
Raw coverage	0.482	0.442	0.390
Unique coverage	0.103	0.063	0.024
Consistency	0.896	0.907	0.944
Solution coverage:	0.580		
Solution consistency:	0.881		

Black circles indicate the presence of a condition, and circles with “X” indicate the absence of a condition. Large circles indicate core conditions and small circles indicate peripheral conditions. Blank spaces indicate “do not care.” Overall, the solution coverage is the total coverage for all configurations.

Among the three configurations, anxiety and daily working hours were deemed necessary conditions because they covered all configurations. This finding indicates that in the sample dataset, nurses who experienced fatigue tended to be those who had worked for a long time and experienced anxiety. Furthermore, the critical explanatory path for nurses’ fatigue accounted for 48.2% of the variance (raw coverage = 0.482; consistency = 0.896). This result indicates that fatigue occurs when nurses are less educated, work longer hours, and experience severe anxiety (i.e., the presence of daily working hours, anxiety, and education: configuration 1), regardless of professionalism and cooperation (i.e., the absence of professionalism and cooperation: configuration 1).

The second explanatory path accounted for 44.2% of the variance (raw coverage = 0.442; consistency = 0.907). This result suggests that regardless of age, cooperation, and professionalism (i.e., the absence of age, cooperation, and professionalism; Configuration 2), anxiety with core conditions, combined with long working hours with edge conditions, leads to significant fatigue (i.e., education and daily working hours; Configurations 2).

The third path that explains nurses’ fatigue accounted for 39.0% of the variance (raw coverage = 0.390; consistency = 0.944), indicating that regardless of the nurses’ age, transition shock, and anxiety level (i.e., the absence of age, transition shock, and anxiety: Configuration 3), a good teamwork atmosphere with core conditions, a low level of professionalism, and long working hours with marginal conditions can lead to the emergence of fatigue (i.e., the presence of daily working hours, cooperation and professionalism: configurations 3).

## 4. Discussion

This study aimed to analyze the factors influencing nurses’ fatigue during COVID-19, including sociodemographic (age and education), psychological (anxiety, transition shock, and professionalism), and work-related variables (collaboration and daily work hours). In addition, this study compared two methods, regression, and fsQCA. Regression allows comparison of the effects of different factors on fatigue, whereas fsQCA allows analysis of the combined effects of different factors on fatigue. This study extends research on factors affecting nurses’ fatigue.

The regression analysis results suggest that greater transition shock, higher anxiety, and poorer teamwork lead to more severe fatigue, while higher professionalism has a mitigating effect. The fsQCA suggested that anxiety is a necessary condition for fatigue. Professionalism, transition shock, teamwork, education, and daily work hours appear to be sufficient conditions for fatigue, findings that are consistent with other studies. Hospitals admit critically ill patients. The potential for viral infection and the long working hours make nurses anxious (14, 35), especially new nurses, transitioning from caring for COVID-19 patients in their previous work department to a department caring for infectious patients. Lack of experience means new nurses lack a sense of control over their work. In addition to working long hours and lacking rest periods, nurses are more likely to experience fatigue. A good working environment has been demonstrated to reduce nurses’ fatigue. Good team relationships indicate smooth communication and rapport among nurses, which helps nurses handle patients more efficiently and enables them to experience psychological support from colleagues, avoid bad feelings, and reduce fatigue. In addition, believing that caring for infectious and critically ill patients is meaningful creates a sense of sanctity and mission for work, which reduces stress and anxiety and reduces fatigue (36).

According to the results obtained from the most important pathway of the fsQCA model, the combination of anxiety, working hours, and education had the most substantial influence on nurses’ fatigue, suggesting that during an epidemic, working long hours while experiencing high anxiety can lead to fatigue. In contrast, individual factors (i.e., education) can also influence fatigue. Studies have demonstrated that anxiety significantly predicts nurses’ fatigue (37, 38). Piper’s fatigue framework emphasizes that psychological states, such as anxiety and depression, are strongly associated with individual fatigue (39) and that nurses’ anxiety leads to a lack of motivation in their lives, decreased concentration, and reduced commitment to their work, which produces fatigue (40). The panic caused by a sudden major epidemic and the lack of family and social support for nurses’ life and work, the resulting anxiety in response to changes in their normal life and work environments, cause severe psychological stress. When internal psychological demands exceed the limits of their ability to cope, individual overload occurs. Nurses may experience increased consumption of personal energy resources and a lack of stress management leading to fatigue. That is, fatigue occurs when nurses cannot manage their stress effectively. Long working hours increase nurses’ anxiety and fatigue, and previous studies have demonstrated that physical work is the most common factor contributing to physical fatigue (41). This finding suggests that fatigue occurs when nurses spend most of their time, energy, and effort on work for long periods, constantly being stretched and failing to take time to rest and relax physically or emotionally (42). Furthermore, a Turkish study showed that nurses’ working hours had a significant influence on anxiety and fatigue (43) and that longer working hours also meant a greater chance of being infected and dealing with complex medical problems or administrative matters. Working hours increased uncertainty about the future, which increased nurses’ fatigue. Thus, working hours bring physical fatigue to nurses on the one hand and exacerbate the adverse effects of anxiety on the other. However, according to Lazarus and Folkman’s cognitive theory of stress, although stressful events influence personal feelings, assessment and coping processes play crucial roles (44). Nurses’ work attitudes affect their perceptions of work stress. Studies show that cognitive level and psychological resilience increase with education (45). More highly educated nurses may view their work positively and have a greater ability to deal with negative emotions, such as anxiety, resulting in less fatigue.

An important feature of this study is that fatigue among epidemic-fighting nurses has rarely been studied from a combinatorial perspective. When univariate analyzes are used, the combined effect of the variables on fatigue is easily overlooked. However, two complementary approaches, regression and fsQCA models were used in the present study. When comparing the two methods, fsQCA complements the regression model by providing multiple pathways in which predictors can be combined in different ways to explain the same outcome. Furthermore, variables that were not statistically significant predictors of fatigue according to the regression analysis (e.g., education and hours worked per day) can affect fatigue when combined with other variables in fsQCA. This finding suggests that fsQCA and regression can be used in combination with different research perspectives.

## 5. Conclusion

The two main findings of this study were that the regression results showed that when anti-epidemic nurses face greater transition shock in a new environment, low levels of professionalism, anxiety, and poor nursing teamwork situations lead to increased fatigue. Second, the fsQCA results showed that anxiety is sufficient for fatigue and that nurses’ educational status, daily working hours, and anxiety are the most effective combination of factors. Therefore, the fsQCA model allows us to consider individual inputs and combinations or interactions between different variables that may lead to a specific outcome. Given the differences between linear relational models and fsQCA, far from prioritizing one technique over the other, the two are complementary and should be used simultaneously in other studies.

## 6. Limitations

One of the main limitations of this study is the limited sample representativeness, including the sampling procedure (non-probability sampling) and geographical location, as this study was based only on hospitals in Heilongjiang Province, China. Although COVID-19 is a global event, stratified probability sampling with different geographic regions could be considered in the future to improve the generalizability of the data.

## Data availability statement

The original contributions presented in the study are included in the article/supplementary material, further inquiries can be directed to the corresponding authors.

## Ethics statement

This study involves human participants and was approved by the Committee on the Ethics of Harbin Medical University (HMUIRB2023017). This study was also conducted by the ethical standards of the Declaration of Helsinki (2008). Moreover, informed consent was obtained from each participant before the start of work. All voluntary participants gave their informed consent with the assurance of confidentiality and anonymity of the data, according to ethical principles for medical research involving human subjects. The database used in this study contains identification data used to protect the privacy of participants.

## Author contributions

HZ, ZhiL, and YF: study design. NW, JuL, JZ, DZ, and CW: data collection. HZ, NW, and XL: data analysis. LL and JiL: study supervision. HZ, ZhaL, and YF: manuscript writing. LL, JuL, and YF: critical revisions for important intellectual content. All authors contributed to the article and approved the submitted version.

## Funding

This study was supported by the National Natural Science Foundation of China (Grant number 71974049).

## Conflict of interest

The authors declare that the research was conducted in the absence of any commercial or financial relationships that could be construed as a potential conflict of interest.

## Publisher’s note

All claims expressed in this article are solely those of the authors and do not necessarily represent those of their affiliated organizations, or those of the publisher, the editors and the reviewers. Any product that may be evaluated in this article, or claim that may be made by its manufacturer, is not guaranteed or endorsed by the publisher.
